# Evaluation of repeated [^18^F]EF5 PET/CT scans and tumor growth rate in experimental head and neck carcinomas

**DOI:** 10.1186/s13550-014-0065-z

**Published:** 2014-12-16

**Authors:** Antti Silvoniemi, Jonna Silén, Sarita Forsback, Eliisa Löyttyniemi, Aleksi R Schrey, Olof Solin, Reidar Grénman, Heikki Minn, Tove J Grönroos

**Affiliations:** 1 Turku PET Centre, Medicity Research Laboratory, University of Turku, Tykistökatu 6A, Turku, FI-20520, Finland; 2 Department of Otorhinolaryngology - Head and Neck Surgery, Turku University Hospital, Turku, FI-20521, Finland; 3 Department of Biostatistics, University of Turku, Lemminkäisenkatu 1, Turku, FI-20520, Finland; 4 Department of Oncology and Radiotherapy, Turku University Hospital, Turku, FI-20521, Finland

**Keywords:** [18F]EF5, [18F]FDG, PET/CT, Hypoxia, Tumor growth rate, HNSCC, Xenograft

## Abstract

**Background:**

Tumor hypoxia is linked to invasion and metastasis but whether this associates with tumor growth rate is not well understood. We aimed to study the relationship between hypoxia evaluated with the positron emission tomography (PET) tracer [^18^F]EF5 and tumor growth. Our second goal was to assess the variability in the uptake of [^18^F]EF5 in tumor between two scans.

**Methods:**

Four human head and neck squamous cell carcinoma (UT-SCC) cell lines were xenografted in flank or neck of nude mice, and tumor size was closely monitored over the study period. The tumors were clearly visible when the first [^18^F]EF5 scan was acquired. After an exponential growth phase, the tumors were imaged again with [^18^F]EF5 and also with ^18^F-fluorodeoxyglucose ([^18^F]FDG).

**Results:**

There was a clear correlation between the percentage of tumor growth rate per day and the [^18^F]EF5 uptake in the latter scan (*r* = 0.766, *p* = 0.01). The uptake of [^18^F]EF5 in the first scan and the uptake of [^18^F]FDG did not significantly correlate with the tumor growth rate. We also observed considerable variations in the uptake of [^18^F]EF5 between the two scans.

**Conclusions:**

The uptake of [^18^F]EF5 in the late phase of exponential tumor growth is associated with the tumor growth rate in mice bearing HNC xenografts.

## Background

Imaging of hypoxia with positron emission tomography (PET) has been the subject of intense research in oncology since hypoxic tumors tend to be more aggressive and have a poorer prognosis than their non-hypoxic counterparts [1]. In particular, poor oxygenation confers radioresistance in head and neck cancer (HNC) [2]. Consequently, there is a great need to develop an appropriate non-invasive method to detect tumor hypoxia before the onset of radiotherapy (RT) of HNC to help in the selection of patients who are candidates for dose escalation protocols of hypoxic tumors [3].

Integrated positron emission tomography and computed tomography (PET/CT) imaging with ^18^F-fluorodeoxyglucose ([^18^F]FDG) is increasingly used in patients with HNC for pretreatment staging and monitoring of therapy response [4]. PET imaging has been postulated to be an ideal method also to detect tumor hypoxia, but one major challenge has been to develop an optimal PET tracer for this purpose [5]. Several PET tracers have been tested recently for the detection of hypoxia, for example 2-(2-nitro-1H-imidazol-1-yl)-N-(2,2,3,3,3-pentafluoropropyl)-acetamide (EF5) labeled with ^18^F-fluorine isotope. [^18^F]EF5 has been evaluated both in preclinical and in clinical studies, and the initial results have been encouraging [6]-[9].

Hypoxic tumors are thought to grow more rapidly than non-hypoxic tumors, because hypoxia induces several biological processes that promote tumor growth, e.g., cell proliferation and angiogenesis [10]. Rapid growth is a general feature of aggressive tumors together with increased metabolism. This can be depicted with PET imaging, although inflammatory reactions may contribute to the signal. In line with this theory, a high uptake of [^18^F]FDG in neoplastic tissue has correlated with tumor growth rate both in experimental and in several solid tumors [11],[12] and is thought to be associated with poor outcome.

While tumor size is another negative prognostic factor in HNC, several studies have attempted to investigate the relationship between tumor size and oxygenation with invasive and non-invasive methods. In a series of classical polarographic studies utilizing pO_2_ histography, it was found that the degree of hypoxia was not related to tumor size [13]. Similarly, the uptake of hypoxia tracer ^18^F-fluoromisonidazole ([^18^F]FMISO) has not been associated with tumor size in several experimental and clinical studies [14]-[16]. In view of these findings and the heterogeneous distribution of hypoxia and proliferation within tumors, it has been deemed necessary to study the extent of the PET tracer based metabolic and hypoxic subvolumes over time. This is due to the current practice of intensity modulated and dose painting techniques which permit the adaptation of volume changes to the initial plan during RT.

Bearing these factors in mind, we aimed to determine the uptake of [^18^F]EF5 and [^18^F]FDG in relation to growth rate in an experimental HNC tumor model. Secondly, we were interested in examining the variability of uptake of the hypoxia tracer over time in an attempt to assess its feasibility for adaptive RT plans where changes in the pattern of uptake have been introduced as a new confounding factor.

## Methods

### Xenografts

Four squamous cell carcinoma cell lines (UT-SCC) established in the University of Turku were used in this study. Different cell lines were utilized to mimic the genetic variability of malignant head and neck tumors in a clinical setting. The characteristics of the cell lines are presented in Table 1. Cells were routinely cultured in Dulbecco’s modified Eagle’s medium (Gibco) containing L-glutamine (Gibco), non-essential amino acids (Gibco), streptomycin, penicillin (Gibco), and 10% FBS (Gibco) at +37°C in a humidified air atmosphere containing 5% CO_2_. For detaching and plating, cells were washed with PSB, trypsinized (Trypsin-EDTA in HBSS, Gibco), and centrifuged at 1,300 rpm for 5 min [7].

**Table 1:** Characteristics of the cell lines

**Cell line**	**Gender**	**Primary tumor location**	**TNM- classification**	**Type of lesion**	**Grade**
UT-SCC 8	Male	Supraglottic larynx	T2N0M0	Primary	G1
UT-SCC 34	Male	Supraglottic larynx	T4N0M0	Primary	G1
UT-SCC 70	Male	Hypopharynx	T4N1M0	Primary	G3
UT-SCC 74A	Male	Mobile tongue	T3N1M0	Primary	G1 to G2

The animal procedures were performed as previously described [7]. Male nude mice (Athymic nu/nu, Harlan, The Netherlands), weighing 28.9 ± 4.0 g (mean ± SD), were total-body irradiated with 4 Gy 1 day before the tumor induction in order to suppress the immune system and facilitate tumor growth. Tumor cells (1−10 × 10^6^) were subcutaneously injected into the flank or neck of each mouse. Mice were maintained in IVC animal cages under controlled pathogen-free environmental conditions (21°C, humidity 55 ± 5%, and lights on from 6:00 a.m. to 6:00 p.m.) with free access to water and standard food. The animals were observed on a daily basis and tumor sizes were closely monitored over the study period (*V* = (π/6) × a × c × b). The starting and end points of exponential growth periods were determined from the tumor growth curves. The percentage of tumor growth rate per day from the exponential growth period was calculated. The experiment procedures were reviewed by the local Ethics Committee on Animal Experimentation of the University of Turku and approved by the Provincial State Office of Western Finland.

### Synthesis of PET tracers

[^18^F]EF5 was synthesized as previously described [8]. The specific activity of [^18^F]EF5, decay corrected to the end of synthesis, exceeded 3.7 GBq/μmol. Radiochemical purity was higher than 98.5% in every production batch.

[^18^F]FDG was synthesized from mannosyl triflate using a nucleophilic method. Radiochemical purity exceeded 95% in every production batch.

### PET/CT imaging

The tumors were clearly visible when they were first imaged with [^18^F]EF5. Mice were anesthetized with 2.5% isoflurane, and body temperature was maintained using a heating pad. PET/CT scans were performed with the Inveon multimodality PET/CT scanner (Siemens Medical Solutions, Knoxville, TN, USA). Following a transmission scan for attenuation correction using the CT modality, an emission scan was acquired in the 3D list mode with an energy window of 350 to 650 keV. Dynamic 80 min long scans were acquired. Sinograms were framed into 25 frames: 6 × 10 s, 4 × 15 s, 2 × 30 s, 2 × 120 s, 1 × 180 s, 6 × 300 s, and 4 × 600 s and reconstructed with an OSEM two-dimensional iterative algorithm. A second [^18^F]EF5 scan and an [^18^F]FDG scan were performed on consecutive days after the clear exponential growth period of tumors. Mice were injected with [^18^F]EF5 (11.1 ± 1.9 MBq) or [^18^F]FDG (10.9 ± 2.8 MBq) (mean ± SD). The mice were sacrificed after the last PET/CT scan and tissue oxygen measurements (see below).

### Data analysis

The Inveon Research Workplace Image Analysis software (Siemens Medical Solutions, Knoxville, TN, USA) was used to analyze the PET/CT images. The images were summed from 60 to 80 min post injection, and the tumors were delineated in the images using the CT image as an anatomical reference. Volumes of interest (VOIs) were drawn over whole tumors and then transformed to the PET image. Radioactivity uptake was calculated as the percentage injected dose per gram tissue (%ID/g) in the whole tumor. In addition, the hottest cluster containing voxels with the highest uptake in 10% of the tumor volume (HC 10%) was determined using the same uptake unit (%ID/g).

### Direct tissue oxygen measurements

Licox® (GMS, Kiel-Mielkendorf, Germany) system was used for direct tissue oxygenation measurements with the Licox CC1.P1 probe with temperature sensor being used in this study. It was 0.65 mm in diameter and had an 18-mm^2^ oxygen sensitive area, which produced an estimate of nearby tissue oxygen tension (Integra Neuroscience). Two of the tumors were measured. Mice were anesthetized while performing these measurements in a similar way as when they were subjected to imaging. Subsequently, a venous cannula was inserted into the centre of the tumor, and the probe was inserted retrogradely through the cannula, which was then removed. The investigator followed continuously the location of the probe and the body temperature of the mouse during the whole recording time. The recordings were continued until the tissue oxygen tension (p_ti_O_2_) level remained stable for at least 20 min.

### Statistical methods

Variables are reported as medians and interquartiles, unless otherwise stated. Pearson correlation coefficients were used to correlate PET parameters (the [^18^F]FDG scan and the first and second [^18^F]EF5 scans) with the percentage of tumor growth rate per day. In addition, Pearson correlation coefficient was used to correlate the tumor volume and the uptake of [^18^F]EF5. *P* values less than 0.05 were considered statistically significant (two-tailed). The statistical analyses were generated using SAS software, version 9.3 for Windows (SAS institute, Cary, NC, USA).

## Results

### Tumor growth

**Figure 1:**
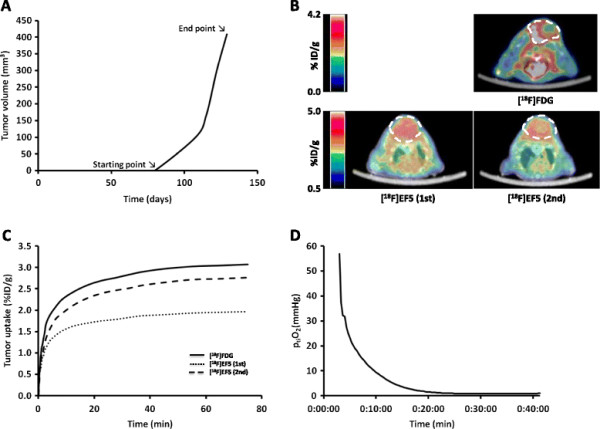
**Growth curve, PET data, and partial p**_**ti**_**O**_**2**_**measurements from tumor nr 5. (A)** Growth curve of the tumor with the starting point and end point of the exponential growth period. **(B)** Axial PET/CT images of the tumor. **(C)** Time-activity curves of the tracer uptake into the tumor in the [^18^F]FDG scan and in the first and second [^18^F]EF5 scan. **(D)** Measurements of the partial pressure of oxygen in the tumor.

There was a considerable variability in growth rates of the individual tumors (Table 2). During the exponential growth phase; the median percentage of tumor growth rate per day was 8.45 (6.64 to 11.92). The median tumor volume at the time of the last scan was 321 mm^3^ (183 to 569 mm^3^). An example of the tumor growth curve is presented in Figure 1A.

**Table 2:** **[**^**18**^**F]FDG and [**^**18**^**F]EF5 uptake values, tumor volumes, and tumor growth rates**

**Tumor nr**	**Cell line**	**[**^**18**^**F]FDG uptake (%ID/g)**	**[**^**18**^**F]FDG uptake (HC 10%)**	**First [**^**18**^**F]EF5 uptake (%ID/g)**	**First [**^**18**^**F]EF5 uptake (HC10%)**	**Second [**^**18**^**F]EF5 uptake (%ID/g)**	**Second [**^**18**^**F]EF5 uptake (HC 10%)**	**First and second [**^**18**^**F]EF5 scans time interval (days)**	**First [**^**18**^**F]EF5 tumor volume (mm**^**3**^**)**	**Second [**^**18**^**F]EF5 tumor volume (mm**^**3**^**)**	**Tumor growth rate (%/day)**
1	8	1.59	2.15	0.54	0.90	0.99	1.60	26	29	183	8.20
2	8	1.31	2.21	1.41	3.45	0.68	1.03	26	91	685	8.69
3	8	1.51	2.31	1.39	2.86	0.95	1.15	26	45	275	7.38
4	34	1.80	2.99	2.35	3.15	2.42	2.89	5	325	367	14.08
5	34	3.06	4.44	1.96	2.56	2.75	3.64	7	330	408	11.92
6	74A	2.23	3.71	1.30	1.80	1.75	2.56	36	223	660	6.64
7	74A	2.76	3.89	0.88	1.30	2.67	3.34	14	53	105	12.08
8	70	1.06	2.89	1.50	3.18	1.37	2.62	15	340	569	6.11
9	70	1.70	5.13	1.39	2.17	1.03	2.15	35	93	274	5.29
10	70	4.58	10.89	2.16	3.28	1.62	3.39	35	30	168	9.17
	Median	1.75	3.35	1.40	2.71	1.50	2.59	26	92	321	8.45
	(Q1-Q3)	(1.51 to 2.76)	(2.31 to 4.44)	(1.30 to 1.96)	(1.80 to 3.18)	(0.99 to 2.42)	(1.60 to 3.34)	(14 to 35)	(45 to 325)	(183 to 569)	(6.64 to 11.92)

### Uptake of PET tracers in xenografts

**Figure 2:**
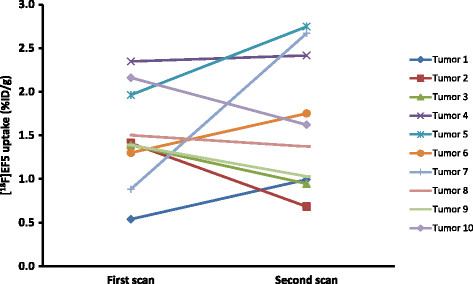
**Whole tumor uptake of [**^**18**^**F]EF5 in the repeated PET/CT scans.**

The uptake of [^18^F]EF5 and [^18^F]FDG in the tumors is presented in Table 2. Figure 1 shows typical PET/CT images and time-activity-curves of one tumor. The median whole tumor uptake for [^18^F]FDG was higher (1.75% ID/g) than for [^18^F]EF5 (first and second scans: 1.40% ID/g and 1.50% ID/g, respectively). There were considerable variations between the repeated [^18^F]EF5 scans. As seen in Figure 2, the uptake in the tumors could either increase or decrease during the tumor growth.

**Figure 3:**
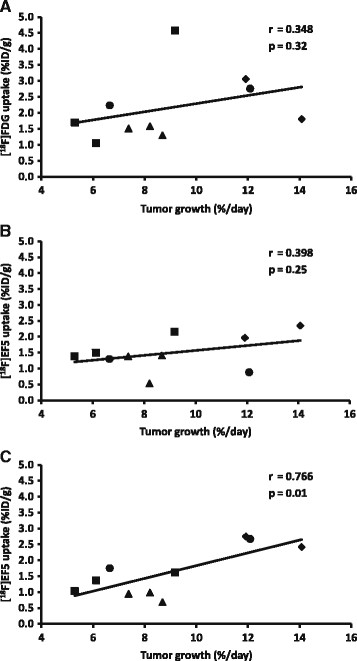
**Whole tumor uptake values compared to the tumor growth rate.** The black line indicates the linear relationship between tumor growth rate and the tracer uptake in the [^18^F]FDG-scans **(A)** and in the first **(B)** and second **(C)** [^18^F]EF5 scans. UT-SCC8 (filled triangle), UT-SCC34 (filled diamond), UT-SCC74A (filled circle), and UT-SCC70 (filled square).

**Figure 4:**
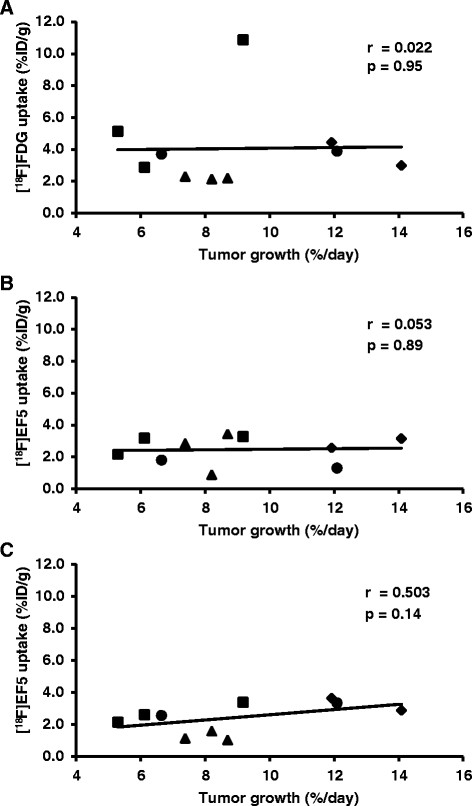
**Hottest cluster (HC 10%) tumor uptake values compared to the tumor growth rate.** The black line indicates the linear relationship between tumor growth rate and the tracer uptake in the [^18^F]FDG-scans **(A)** and in the first **(B)** and second **(C)** [^18^F]EF5 scans. UT-SCC8 (filled triangle), UT-SCC34 (filled diamond), UT-SCC74A (filled circle), and UT-SCC70 (filled square).

The percentage of tumor growth rate per day and [^18^F]FDG uptake seemed to point to a relationship (*r* = 0.348, *p* = 0.32) as did [^18^F]EF5 uptake in the first scan (*r* = 0.398, *p* = 0.25), although neither of the relationships was statistically significant. Instead, the uptake of [^18^F]EF5 in the latter scan showed a stronger correlation with the tumor growth rate (*r* = 0.766) which was statistically significant (*p* = 0.01) (Figure 3). Tumor growth rate was also correlated with the HC 10% uptake of the tumor (Figure 4) for all three scans. The strongest, although not a significant relationship, was seen between the HC 10% uptake of the second [^18^F]EF5 scan and tumor growth rate per day (*r* = 0.503, *p* = 0.14).

There was no correlation in the latter scan between the [^18^F]EF5 uptake and the tumor volume (*r* = −0.217, *p* = 0.55). However, the tumor volume seemed to be in relation with the [^18^F]EF5 uptake in the first scan, when the tumors were smaller (*r* = 0.510, *p* = 0.13), although this correlation did not reach statistical significance.

### Direct oxygen measurements

Two tumors (nr 4 and 5) were measured with Licox system. Both tumors were clearly hypoxic according to their p_ti_O_2_ values, which were 3.6 and 0.9 mmHg, respectively. An example of direct oxygen measurements is presented in Figure 1D.

## Discussion

A high growth rate and hypoxia are the characteristics of HNC with poor prognosis. Certainly, there are many other important features contributing to a low survival rate, e.g., tendencies towards invasion and metastasis [17]. Nonetheless, of these features, tumor growth rate is probably the simplest to measure in experimental studies. In spite of this, there is only limited data on the association between [^18^F]FDG uptake and tumor growth rate in HNC in different experimental systems. Hypoxia has been associated with rapidly growing tumors in general [10], although the level of hypoxia does not usually correlate with tumor volume [13]. As far as we are aware, none of the currently known hypoxia PET tracers have been evaluated to determine how well they reflect relationship with the tumor growth rate.

Another novel perspective in this study was the performance of repeated [^18^F]EF5 imaging with time at different stages of the exponential tumor growth. The intention was to compare the whole tumor uptake of those two scans in order to evaluate the utility of [^18^F]EF5 imaging to detect tumor hypoxia progression in an experimental setting. For practical reasons, e.g., unpredictable tumor growth rates, we could not utilize a constant time frame between the [^18^F]EF5 scans. Instead, we were able to perform the [^18^F]FDG scans and the latter [^18^F]EF5 scans on consecutive days. In addition, we measured the oxygen level with the Licox system. Both measured tumors were found to be hypoxic, and the [^18^F]EF5 uptake values of the latter scan in these tumors were higher than the corresponding median [^18^F]EF5 uptake value (Table 2).

There was a considerable variation between the tumor [^18^F]EF5 uptakes of the two scans, as shown in Figure 2. Probably due to the growth of tumors, some variation was evident between the two scans. It is also well known that the uptake rate of hypoxia PET tracers in tumors may fluctuate over time, probably due to changes in acute hypoxia. Nonetheless, the time scale and pattern for these changes remain poorly understood, and conflicting findings with different tracers in experimental and human studies have been reported. Busk et al. performed an experimental reproducibility study with mice carrying human cervix tumor xenografts. In that study, a high correlation was noted between the two PET scans with the hypoxia tracer ^18^F-labeled fluoroazomycin arabinoside ([^18^F]FAZA) performed on consecutive days [18]. Nehmeh et al. detected considerable variation among repeated [^18^F]FMISO scans performed three days apart in patients with HNC [19], whereas Okamoto et al. reported high reproducibility of [^18^F]FMISO scans conducted 2 days apart in another clinical study with HNC patients [20]. Nevertheless, very little is known about changes in the PET tracer uptake in a hypoxic malignant tumor over a larger time scale and even less is known about the potential relationship of these changes with the behavior of the tumor.

In our study, only a weak positive correlation (*r* = 0.348) was found between the tumor growth rate and the [^18^F]FDG uptake in the late period of exponential tumor growth. Based on our previous knowledge of tumor growth rate and metabolic activity in comparable cell lines [21], the correlation between [^18^F]FDG uptake and tumor growth rate would have been expected to be somewhat stronger. There was only a weak positive correlation (*r* = 0.398) between the [^18^F]EF5 uptake of the first scan and the tumor growth rate, in contrast to the clear correlation (*r* = 0.766) noted between the latter [^18^F]EF5 scan which was performed during the late phase of exponential growth. We also determined the tumor uptakes as HC 10%. Only the HC 10% value in the second [^18^F]EF5 scan seemed to point toward a relationship (*r* = 0.503) with the tumor growth rate. Accordingly, we conclude that the uptakes of [^18^F]FDG and [^18^F]EF5 in the first scan are independent of the tumor growth rate. More rapidly growing tumors showed a higher [^18^F]EF5 uptake at the late period of the exponential growth phase. The biological background for this relationship is obscure since hypoxia itself may drive tumor progression, but rapid cell proliferation in the tumor would be expected to contribute to the development of hypoxia [10].

One limitation of this study is the small and variable tumor volume, particularly in the first [^18^F]EF5 scans. As known, the partial volume effect may lead to underestimation of the detected radioactivity in tumors that are of a size less than three times the full width at half maximum of the reconstructed image resolution [22]. This is a fact that has to be kept in mind when considering further conclusions regarding our results from the first [^18^F]EF5 scans. Nevertheless, at the latter [^18^F]EF5 scan, tumors were larger, and hence, the risk of biases introduced by the partial volume effect is not considered to affect these findings.

The uptake of [^18^F]EF5 in the latter scan did not correlate with the tumor size (*r* = −0.217) in our study population. This is in line with many previously reported studies, which have concluded that there is no connection between tumor size and hypoxia [13]-[16]. Clearly, a large tumor size cannot be used as a surrogate marker of poor oxygenation, and this underlines the fact that similar-sized tumors may need different radiation doses to become eliminated. However, although not statistically significant, we observed a trend toward a relation between the [^18^F]EF5 uptake in the first scan and the tumor volume (*r* = 0.510, *p* = 0.13). This weak relationship might be explained by the partial volume effect in smaller tumors.

Recent studies and reviews on PET/CT imaging of tumor hypoxia have highlighted that there are challenges both in the detection of tumor hypoxia and in the clarification of the relevance of these results. Spatial and temporal variation in tumor hypoxia occurs frequently, but this phenomenon is still inadequately understood. Therefore the future studies on hypoxia PET/CT-imaging should be designed in such a way that the utility of a new tracer or a new method can be evaluated by comparing the imaging data not only to hypoxia-related markers or to direct oxygen measurements of the tumor but also to the behavior of the tumor and treatment outcome [5]. This study demonstrated the initial relationship between [^18^F]EF5 uptake of the tumor and tumor growth rate, but a larger study population would be needed to confirm this observation. It is interesting that this relationship was stronger for a hypoxic than for a metabolic tracer; this may be due to timing of the second measurement when the hypoxic fraction is large.

## Conclusions

We found that the uptake of the hypoxia tracer [^18^F]EF5 in the late phase of exponential tumor growth is associated with the tumor growth rate in mice carrying HNC xenografts. A considerable variation in the uptake of [^18^F]EF5 was also detected between the two scans. The temporal variation of tumor hypoxia remains a poorly understood phenomenon.

## Competing interests

The authors declare that they have no competing interests.

## Authors’ contributions

AS contributed to the tumor induction, PET/CT imaging, direct tissue oxygen measurements, analysis and interpretation of the data, and preparation of the manuscript. JS contributed to the cell culturing, tumor induction, PET/CT imaging, and preparation of the manuscript. SF and OS contributed to the synthesis of the tracer. EL contributed to the statistical analysis of the study data. ARS contributed to the direct tissue oxygen measurements. RG contributed to the study design and supervision of cell culturing. HM contributed to the study design and interpretation of the data and preparation of the manuscript. TJG contributed to the conception and design of the study, to the experimental procedures, to the analysis and interpretation of the data, as well as to the preparation of the manuscript. All authors read and approved the final manuscript.
